# Experiences of Advanced Health Assessment Simulation Based on the Education Needs of Gerontological Nurse Practitioner Students

**DOI:** 10.3390/healthcare11081162

**Published:** 2023-04-18

**Authors:** Jiyoung Kim, Hyunju Dan

**Affiliations:** 1 Department of Nursing, Inha University, Incheon 22212, Republic of Korea; 2 Department of Nursing, Gangdong University, Eumseong-gun 27600, Republic of Korea

**Keywords:** advanced health assessment, graduate students, nursing education, nurse practitioner, simulation

## Abstract

There is an insufficient exploration of how simulation educational approaches improve the job performance of students in gerontological nurse practitioner (GNP) courses. To increase the effectiveness of simulation education in GNP courses, it is necessary to explore the advanced health assessment simulation curriculum. This study aimed to investigate GNP students’ educational experiences with the advanced health assessment simulation program by reflecting the needs of GNPs. A qualitative study design was employed for this study; focus group interviews were conducted among eight GNP students who participated in this simulation program. The focus group interview identified three theme clusters: ‘a high-fidelity simulator that reproduces a real-life setting’, ‘experience with standardized patients as a reference for normal older people’, and ‘application in the clinical field’. Through simulation education, GNP students were able to safely demonstrate knowledge and use what they learned for clinical practice. The development and utilization of simulation education for the GNP program would help to enhance the clinical competency of students.

## 1. Introduction

Simulation has become an important educational approach in nursing education and is expected to increase direct nursing practice opportunities and reduce the risk to patients by implementing an environment similar to the actual clinical setting [1,2]. Simulation education has been reported to have a positive impact on patient health, such as reducing morbidity and/or mortality outcomes [3]. However, simulation education approaches for graduate students or nurses have not been explored sufficiently compared to research on undergraduate students, and the usefulness of simulation in advanced nursing practice (ANP) courses have not been evaluated fully [1,2,4]. Utilization of an advanced practice nurse (APN) simulation education suitable for the field of activity in several countries was proposed previously [5].

APNs must demonstrate complex decision-making skills and clinical capabilities suitable for expanded practice based on outstanding knowledge [6]. ANP in Korea emerged as a government policy to provide health care services to disadvantaged people and to realize equal health care rights. From 2005 to 2020, a total of 7890 APNs were produced in 13 fields; among them, gerontological nurse practitioners (GNPs) were the most numerous at 2429. This action was necessary to limit the rising medical costs associated with the increasing aged population [7]. Although it is a critical role of the GNP to provide a systematic assessment of changes in the health status of older people in Korea, the APN program is centered on lectures and is criticized for lack of practical education [8].

One study revealed that health assessment practices for APN students in Korea are based mainly on general observations. The compulsory advanced health assessment training in the APN program was not utilized routinely in clinical practice, as it can act as a barrier to early detection and intervention of potential health problems of individuals, which is the main role of APNs. Ninety-three percent of survey respondents answered that in-depth, advanced health assessment education was necessary. In addition, to improve the health assessment ability of APNs, practice-oriented education based on educational needs is needed [9]. Therefore, the provision of a simulation program is necessary to enable GNPs to increase their specialized skills and competency. In particular, simulation education for GNP students is expected to contribute to clinical relevance and continuity of education for older people.

The high-fidelity simulator (HFS) used in simulation education can replicate a multitude of clinical scenarios. The learning experience provided by these simulators is consistent [10]. A standardized patient (SP) is an innovative method to improve the trainee’s communication skills without harming patients [11]. The SP provides nonverbal signals such as body language, facial expressions, and physical exercise [12] and feedback on the trainees’ communication skills and attitudes [13]. Simulation education using both HFS and SP and integrating the advantages of each type enhances learner confidence [11]. Staples and Pierazzo [10] developed simulation scenarios for adult, older, and child subjects for nurse practitioners and confirmed the positive effect of applying multi-modal simulation. In the survey results on the use of simulation in APN programs in the United States and Canada [2], SPs (68%), unfolding case studies (66%), and role play (61%) were used most commonly. There have been insufficient studies to identify the effect of education using both HFS and SPs. Simulation education, which incorporates HFS and SP, is considered to be a useful method for improving advanced nursing practices. We suggest the incorporation of simulation education into the curriculum of GNPs.

The purpose of this study was to explore GNP students’ educational experiences with the advanced health assessment simulation program that integrates HFS and SP by reflecting the needs of GNPs.

## 2. Materials and Methods

### 2.1. Study Design and Participants

A qualitative research method was conducted to apply the advanced health assessment simulation program to GNP students and to investigate the educational experience of students with the program. The participants were recruited by purposive sampling [14]. In the study, the target population was 210 Korean graduate students from 25 educational institutions with GNP courses. The accessible population comprised graduate students of two universities among the total educational institutions. The inclusion criteria for participants were GNP students who are current in-service nurses and are taking or have taken within the last two years advanced health assessment courses. GNP students who did not work in hospitals or who communicated poorly were excluded. A minimum of five samples was designated from previous studies conducted on 5–7 participants [12,15]. Eight students participated in this study until the theoretical saturation of the data. Participants with various work types, areas, and careers were recruited to obtain different perspectives and opinions while maintaining group homogeneity in sampling. The age range of the focus group interview participants was 28–52 years, and all were women. Their total clinical experience ranged from five years and two months to twenty-three years (Table 1).

### 2.2. Procedure

This study attempted to develop the simulation scenario considering the elements necessary for the design of simulation education presented by The International Nursing Association for Clinical Simulation and Learning (INACSL) [16]. These elements include needs assessment, measurable objectives, simulation format, clinical scenario or case, fidelity, facilitator/facilitative approach, briefing, debriefing and/or feedback, evaluation, and participant preparation.

We investigated the need for advanced health assessment simulation among 14 in-service nurses who graduated from or completed the GNP program. The self-reported education needs questionnaire was organized based on a literature review and contents of the standard education courses for advanced health conditions presented by the Korea Accreditation Board of Nursing [17]. GNP nurses evaluated the degree of simulated training needs using an HFS and SPs through a five-point Likert scale. Basic physical assessment techniques, including history-taking, inspection, auscultation, palpation, and percussion, were identified as the highest necessity for HFS. Evaluation of cardiovascular and gastrointestinal systems was also given high priority. GNP nurses suggested using the simulations to assess abnormal symptoms. The precedence for SP use was: major symptoms detection, basic physical examination techniques, and neurologic system assessment. The highest needs centered on systems characteristically different between the elderly and adults.

We decided to develop 2 unhealthy elderly case scenarios using an HFS (SimMan 3G) and 1 healthy elderly case scenario using SPs. Scenarios on the selected topics were developed by the first author, a GNP in Korea, and co-author, a family nurse practitioner. Unhealthy elderly cases using the HFS were completed by adding vital signs and imaging examinations to suit the situation. The learning objectives were set to assess the health status and needs of the subjects and to perform the physical examination assessments by each system. In addition, the objectives were set to record and manage the collected data and to cultivate trainee competence in identifying health problems by distinguishing between normal and abnormal conditions. SPs were four female senior citizens in their 80s who had seven years of experience participating in graduate student health assessments. These women were selected as healthy, asymptomatic elderly people taking medication for chronic diseases such as hypertension or osteoarthritis. Researchers provided the patients with the information on the contents of the health assessment class in advance.

The simulation education was designed as a separate non-regular course. This simulation education consisted of pre-briefing, simulation running, and debriefing (Table 2). The researchers were also facilitators, actively guiding learners to understand and reflect on the situation.

### 2.3. Data Collection

Focus group interviews were conducted to allow participants to share their views and experiences with the advanced health assessment simulation program. One researcher who was not a professor at the university led the focus group interviews and guaranteed the confidentiality of those who participated in the research. The data collection was conducted from December to January 2019, one to two weeks after simulation education. The focus groups in this study were made up of eight students who participated in the advanced health assessment simulation program, divided into two groups, and four members each [18,19]. There were two face-to-face focus group sessions, each lasting approximately 50 min. All interviews were recorded with the consent of the participants. All observations during the interview, such as facial expressions, behaviors, voice tone, degree of silence, and emotions of the group, were recorded in a field observation log. The researchers asked open-ended questions such as, “How was the experience of simulation education for the advanced health assessment?”, “What did you learn from the experience of simulation education for the advanced health assessment?”, “What did you realize through the experience of simulation education for the advanced health assessment?”, and “What did you feel you did well?” After the interview, participants were asked to write a reflective essay, allowing for the organization of experiences in more depth through reflection.

### 2.4. Data Analysis

All interviews were recorded and hand-transcribed verbatim by one researcher who directly participated in the focus group interviews; the transcripts were reviewed by both researchers to enhance accuracy. The collected data were analyzed using the qualitative content analysis method in accordance with the preparation, organization, and reporting phases presented by Elo and Kyngäs [20]. The Excel program was used to structure the collected data more efficiently. Two researchers carefully discussed the descriptive data and conducted qualitative content analysis together. During the preparation phase, meaningful words (analytical units) were identified and coded by repeatedly reading descriptive data. In the organizational phase, the similarities and differences of analytical units were compared and analyzed to form theme and theme clusters. The final phase was to report the results produced. Results of the qualitative content analysis were validated by repeated reading and testing and by confirmation by participants and qualitative researchers. In addition, in order to evaluate the rigor of this study, we confirmed the study in four aspects: true value, applicability, consistency, and neutrality suggested by Sandelowski [21]. For true value, the authors explained to the participants that their statements were not a part of their individual grades and ensured the data validity by receiving participant confirmation of their original statements. For applicability, the selection criteria were graduate students with various work types and careers and the ability to express experiences. In addition, the results of this study were presented to two GNP students who met the selection criteria but did not participate in the research to ensure suitability. For consistency, we followed the content analysis method of Elo and Kyngäs [20], and we collected and analyzed the data directly in all collection and analysis processes. For neutrality, assumptions and understanding of research phenomena were discussed in advance. Efforts were made throughout the study to minimize bias and to accurately reflect the participants’ experiences and views.

### 2.5. Ethical Considerations

The research received institutional review board (IRB) approval from the researcher’s university. Participants were given explanations of the purpose, methods, and confidentiality of the study. The study was conducted after obtaining voluntary written informed consent from the participants.

## 3. Results

This study identified six themes and three theme clusters from eight participants. The three theme clusters were ‘a high-fidelity simulator that reproduces a real-life setting’, ‘experience with standardized patients as a reference for normal older people’, and ‘application in the clinical field’.

### 3.1. A High-Fidelity Simulator That Reproduces a Real-Life Setting

#### 3.1.1. Confidently Identifying Normalities and Abnormalities Related to the Patient Condition in Virtual Situations

Participants were able to compare and analyze clinical symptoms by performing the same technique that they had learned in class through the simulation education. These experiences helped participants to compare the normal and abnormal health conditions of older people.

*In the clinical field, if abnormalities were the concern, we were missing the normal sounds for comparison, but simulation education can compare normal and abnormal sounds, which will help clinical nursing.*

(P4)

In addition, simulation allowed participants to practice with much less stress than in real-life urgent situations, and they expressed the ability to actively perform in real clinical practice through such virtual situation.

*Due to the urgent situation in the real clinical field, the patient’s symptoms were poorly checked through inspection, palpation, or auscultation; but the simulation education allowed the patient to observe the symptoms.*

(P7)

#### 3.1.2. The Limits of the Simulator

The participants reported the limitation that the HFS was not able to provide non-verbal signals, such as physical cues or facial expressions, to help to make a treatment decision. They also said that the absence of an actual person in simulation education using an HFS was a limitation in assessing health.

*We cannot assess cerebellum functions such as equilibrium sense and motor nerves. This part is pity.*

(P5)

### 3.2. Experience with Standardized Patients as a Reference for Normal Older People

#### 3.2.1. Experiencing Emotional Interactions with Standardized Patients despite Difficulties in Verbal Communication

Participants expressed difficulties in communicating with older SPs who repeated the same words or discussed topics unrelated to the questions asked. SPs spent significant time explaining their situation, and participants had to be more focused to achieve their educational goals within a set time frame.

*She tended to talk about her situation constantly… Getting all the information in a short time was difficult.*

(P1)

Nevertheless, participants experienced emotional responses during simulation training with SPs, who were real people and were able to develop an attitude of concern toward the individual. Participants said that this experience allowed them to learn how to communicate with older people and to focus on practice. They also reported different interactions with older people when using HFS simulation education compared to SP education.

*I made the checkup sequence and flow naturally so that the subject did not feel uncomfortable and provided emotional support so as not to be awkward or unfamiliar and anxious.*

(P1)

#### 3.2.2. Recognition of Normal Aging

Participants reported that older SPs had no clinically significant findings caused by aging. They felt the need to be familiar with normal aging to prevent clinical judgment errors. They also said that SP education demonstrated the importance of knowledge regarding the normal aging process.

*The subject did so well. Because she didn’t have any pain, so I think I learned more. She didn’t have pain, and she could do full ROM (range of motion). Limits on what is normal need to be established if someone is worse than this grandmother. I think that this woman’s condition has become a reference point and think I can remember this reference point when keeping the standard patient in perspective.*

(P6)

### 3.3. Application in a Real Clinical Field

#### 3.3.1. Knowledge and Skills Transfer to the Clinic

Although the conventional curriculum had limitations in the conveyance of theoretical information as actual information, participants acquired useful professional knowledge through an advanced health assessment simulation program. Participants felt that they were equipped with the necessary competencies for practice after integrating and training different theories and practices through simulation education.

*Comparing and analyzing what was learned in theory with direct inspection of clinical symptoms by palpation, percussion, and auscultation was educational.*

(P7)

Participants experienced the transfer of advanced health assessment knowledge and skills from simulation education to clinical fields. They utilized basic physical examination techniques such as auscultation and percussion in clinical practice and applied knowledge and techniques for each body system.

*When I palpated and percussed the patient’s abdomen in Intensive Care Unit (ICU), I discovered that the patient now needs paracentesis. The patient’s feeling a little stuffy… When I performed the exam and palpated his abdomen, he had distension that was different from normal… At first, percussion worked best for ascites patients. Percussion was very helpful when assessing. Even if I had time before, I wouldn’t think of percussion, but now I do.*

(P4)

#### 3.3.2. Utilization within the Role of GNPs

Participants thought that the advanced health assessment simulation program provided an opportunity to consider the role of GNP and the scope of work. They visualized the performance of roles such as collecting disease-related data, interviewing older people for examination, and evaluating detailed and comprehensive functions.

*Performance of preliminary examinations by GNPs would be helpful. Nurse practitioners need to do thorough assessments. Right now, I direct first-time stomach ache patients to the internal medicine doctor. But I think that the nurse practitioners would be better utilized by performing advance assessments, including those for pain, and providing doctors with written results of these assessments.*

(P6)

## 4. Discussion

This study explored the educational experiences of GNP students with the advanced health assessment simulation program. Quality content analysis of the advanced health assessment simulation program experience through a focus group interview resulted in three theme clusters. The first theme, ‘a high-fidelity simulator that reproduces a real-life setting’, allowed participants to identify normal and abnormal conditions of the subject through advanced health assessment simulation education. Our findings supported the results of a systematic review of the effectiveness of simulation education using an HFS in an advanced nursing program that HFS simulation education was more effective in increasing students’ knowledge and skills than were other methods, such as online learning and traditional classroom lecture [22]. In a study by Tuzer et al. [11], nursing students reported that heart and lung sounds in simulation education using an HFS were clear and distinguishable. Participants also noted a sense of security with virtual situations, seeing these as a safe environment in which to practice communication and health examination skills. Mistakes made during simulation training can be teaching and learning opportunities that do not result in harm to patients [10]. However, simulation education has limitations; simulators differ from reality even with the maximum reproduction of an actual patient. This is consistent with the finding of limited physical characteristics of the simulator [23], as well as the finding that simulation education was insufficient to simulate a real patient’s heart murmur [24]. GNP students can improve their health assessment skills and competency through repetitive practice in a controlled and low-pressure environment using simulation education with an HFS. Therefore, simulation education should be used actively in APN courses. Although advanced health assessment simulation education was used as a realistic representation of observation-oriented clinical practice, additional opportunities are needed to impart clinical experience to overcome the simulator limitations when designing advanced health assessment simulation education.

The second theme was ‘experience with standardized patients as a reference for normal older people’. Participants complained of difficulties in communicating with older people who participated in simulation education as SPs. Our results were comparable with those of a previous study that examined communication with older people. Lee and colleagues [15] reported that nurses who cared for hospitalized older people expressed the importance of communicating with patients at their level and of education on how to communicate and respond to older patients. One participant (P6) expressed that building a rapport by meeting and talking to older people in groups would be helpful before the simulation education. Unfortunately, the time constraint of one hour for exploring chief symptoms and health assessment hinders effective communication. Therefore, providing sufficient time and opportunity for the formation of mutual trust is necessary to utilize simulation education with SPs as a learning strategy for improving communication. Participants experienced emotional interaction through simulation with SPs. This is similar to the finding of previous research that SPs in simulation education had emotional responses [11,12]. In addition, simulation education with SPs increased participant awareness of normal aging processes. Gerontological nursing education often focuses on diseases, while specialized knowledge and skills regarding the characteristics of older people are important to provide optimal geriatric health care [15]. Our findings were consistent with those of a prior study [25] in assessing SPs; students required more critical thinking than when using an HFS. Participants were able to focus better and improve critical thinking solutions when evaluating SPs compared to HFS. Knowledge of normal physical changes in the aging process is the basis for understanding geriatric diseases as well as for identifying and interpreting abnormal symptoms. Exploring the research and educational potential of simulation education programs for a variety of subjects is necessary.

The final theme, ‘application in the clinical field’, explained that theoretical knowledge could be applied in practice through simulation education. Through training in skills required for clinical practice, we were able to transfer knowledge and skills to use in clinical sites for actual, advanced health assessments. In particular, the transition to the clinical field is important for confirming the clinical usefulness and value of GNP simulation education. A study of undergraduates showed improvements in pre-clinical skills, facilitation of the learning process, and improved communication skills [11] but could not confirm knowledge transition to the clinical field. Given the lack of instructors, restrictions on patient access in clinical practice, and decreases in clinical field exposure [26,27], high-quality simulation education is critical to APN education. Education on case studies and objective structured clinical examination (OSCE) was reported by GNPs to be difficult to translate into actual performance [28]. Additional research investigations to confirm the transfer of knowledge, skills, and attitudes from simulation to the clinical field support the importance of simulation in APN education [13]. Therefore, based on the results of this study, simulation education should be utilized in GNP education as a learning strategy for the transfer of knowledge and skills to the clinical field. Furthermore, participants commented on the use of GNPs’ advanced health assessment skills within the scope of practice. To promote the nurse practitioner system in Korea, the utilization of APNs to maximize their effectiveness is necessary and will augment healthcare professional utilization in the clinical field [8]. In particular, for long-term care hospitals in Korea, without 24 h residential doctors, APNs can be especially effective. In these settings, APNs can use their knowledge to assess and diagnose potential health problems, including current diseases [29]. Therefore, the provision of a high level and quality of nursing performance within the scope of GNP practice is a high priority. Advanced health assessment knowledge and techniques learned through simulation education are necessary to achieve such a level of performance.

This study analyzed GNP students’ educational experiences with the advanced health assessment simulation program. Potential limitations should be considered when interpreting our study results. The first is that this study was implemented at two institutions, and the number of participants was small. In future research, we will need to secure research participants in multiple centers and repeat the study. In addition, since the advanced health assessment simulation program is designed separately as a non-regular course, further research into its integration into the curriculum and verification of its performance needs to be conducted. Finally, this study implemented simulation education for advanced health assessments, but further studies are needed to develop and verify the effectiveness of simulation education related to the clinical practice of GNPs.

## 5. Conclusions

This study sought to experience a comprehensive and systematic health assessment simulation education program by reflecting the needs of GNPs. The results of this study are noteworthy in that they provide a basis for the development and application of an advanced health assessment simulation of the education program for gerontological nurse practitioners. The education was conveyed using both a high-fidelity simulator and standardized patients, which reflected the reality and needs of the clinical field. Participants were able to reliably identify the normal aging processes and abnormal age-related conditions of the subjects. Participants were also able to use their knowledge and skills acquired through simulation education in clinical practice. Meanwhile, there have been many studies on simulation education among nursing college students and nurses in Korea, but there have been few studies on student experiences in a GNP course. This study is the first to explore simulation education experiences and confirm their effectiveness among GNP students in Korea. Although the results of this study included a small number of GNP students, they suggest valuable considerations regarding suitable simulation of the characteristics of the target older people and can be helpful in the development and retention of advanced health assessment knowledge and skills necessary for successful clinical practice. Furthermore, a more accurate health assessment can foster the accuracy of nursing diagnosis and high-quality nursing care for older people. It is necessary to develop various simulation programs and verify their effectiveness for active use in GNP courses. Further research on simulation program development and student experiences is needed to improve the professional nursing competency of students in APN courses.

## Figures and Tables

**Table 1:** Characteristics of Participants (N = 8).

No.	Age (yr)	Gender	Marital Status	Work Type	Work Area	Total Clinical Career	Clinical Career in Present Unit
1	47	Female	Married	manager	Long-term care ward	23 years	1 year and 2 months
2	45	Female	Married	manager	Long-term care ward	19 years and 10 months	3 years and 6 months
3	45	Female	Married	nursing staff	Surgical ward	10 years	2 years
4	32	Female	Single	shift work	Intensive care unit	10 years	10 years
5	52	Female	Married	manager	Infection control office	20 years and 9 months	7 years and 4 months
6	32	Female	Married	nursing staff	Outpatient	10 years	2 years
7	28	Female	Single	shift work	Medical ward	5 years and 2 months	4 years and 2 months
8	37	Female	Single	nursing staff	Operating room	16 years	15 years

**Table 2:** Overview of the Developed Simulation Course.

Day	Time	Scenarios Contents	Practice Contents
1: Unhealthy elderly 2 case: A high-fidelity simulator	30 min	Pre-briefing	
	10–20 min per group	Simulation Running—Case 1 scenario: 72-year-old man with heart failure: weight loss for 2 months and diarrhea 2 days ago, reduced tactile vibration and dullness in the percussion of the right chest (provided with vital signs and a chest X-ray of the pleural effusion finding).	- Exploring major symptoms - Radiographic image reading - Pulse palpation - Auscultation of heart sound, breathing sound, and bowel sound - Glasgow Coma Scale assess - Assessment of pupil size and light reflex - Communication and recording
	10–20 min per group	Simulation Running—Case 2 scenario: 65-year-old man with chronic obstructive pulmonary disease: dyspnea and hypoxia after abdominal surgery (vital signs, oxygen saturation, and chest X-ray provided)	
	30 min	Debriefing	
2: Healthy elderly case: Standardized patients	1 h per group	Simulation Running Scenario: 80-year-old woman	- General assessment - Physical measurement, vital signs, pain assessment - Head-to-toe physical examination including head, face, eyes, ears, nose, mouth and throat, neck, skin, lungs, heart, abdomen, upper and lower extremities, nervous system, musculoskeletal system (using examination instruments such as ophthalmoscope, rhinoscope, and otoscope) - Korean version of Mini-Mental State Examination for Dementia Screening (MMSE-DS) - Communication and recording
	30 min	Debriefing	

## Data Availability

Data analyzed in the current study are available from the corresponding authors on reasonable request.

## Abbreviations

GNP	Gerontological Nurse Practitioner
ANP	Advanced Nursing Practice
APN	Advanced Practice Nurse
SP	Standardized Patient
HFS	High-fidelity Simulator
